# Different dietary starch patterns in low-protein diets: effect on nitrogen efficiency, nutrient metabolism, and intestinal flora in growing pigs

**DOI:** 10.1186/s40104-022-00704-4

**Published:** 2022-06-02

**Authors:** Junyan Zhou, Lu Wang, Lijie Yang, Guangxin Yang, Xiangfang Zeng, Shiyan Qiao

**Affiliations:** 1 State Key Laboratory of Animal Nutrition, China Agricultural University, Beijing, 100193 People’s Republic of China; 2 Beijing Bio-feed additives Key Laboratory, Beijing, 100193 People’s Republic of China

**Keywords:** Intestinal flora, Low-protein diet, Nitrogen efficiency, Nutrient metabolism, Starch patterns

## Abstract

**Background:**

Protein releases amino acids faster than starch releases glucose in digestive tract of pigs fed low-protein (LP) diets. Poor synchronization of dietary glucose and amino acids supply leads to compromised nitrogen efficiency. Dietary starch patterns modulation may improve this situation.

**Methods:**

Growing barrows (29.7 ± 2.0 kg) were randomly allotted into 5 dietary treatments with LP diets consisting of different purified starches. Treatments included: waxy corn starch (W LP), corn starch + waxy corn starch (C + W LP), corn starch (C LP), pea starch + waxy corn starch (P + W LP) and pea starch (P LP). In the experiment, growth performance, protein deposition, nutrient metabolism, and fecal microbial community of pigs were investigated. In vitro starch digestion was used for predicting the in vivo glucose response.

**Results:**

Dietary starch in vitro glucose release profile was determined by starch source and the ratio of amylopectin and amylose. C + W LP treatment showed decreased total nitrogen excretion and plasma citrulline concentration and improved plasma leptin concentration among treatments (*P* < 0.05). Besides, the highest nitrogen apparent biological value, whole-body protein deposition and growth performance and lowest urinary nitrogen excretion were also observed in C + W LP treatment. Compared with the other groups, C + W LP and C LP showed increased plasma pyruvate, IGF-1, and lipase concentrations (*P* < 0.05). The W LP group presented dramatically increased plasma alanine and urea nitrogen concentration and decreased aldolase and leptin concentrations (*P* < 0.05). Dietary starch patterns did not make an impact on bacterial richness and diversity, but changed the taxonomic and functional structures of the microbial communities. Microbial protein fermentation product (isobutyrate and isovalerate) presented increased in P LP treatments compared with the other treatments (*P* < 0.05).

**Conclusions:**

Dietary starch patterns modulation can regulate dietary glucose release profile, nutrient metabolism, protein turnover, and fecal microbial fermentation in pigs. The optimal dietary glucose release profile effectively strengthened whole-body protein deposition and improve nitrogen efficiency and growth performance in growing pigs fed LP diets.

## Introduction

Low-protein (LP) diets, supplemented with crystalline amino acids, can precisely satisfy the amino acids requirements of animals, and is considered the key to efficient production of animal husbandry [1, 2]. Crystalline amino acids exist in the form of amino acid monomers, which can be absorbed by intestinal cells into the blood or oxidized, as soon as they enter the intestine [3]. While dietary digestible starch can release glucose only after a period of gastrointestinal digestion. Therefore, compared with starch-derived glucose, the crystalline amino acids-derived amino acids in the diet can be released much faster.

Consequently, low protein diets supplemented with large amounts of crystalline amino acids induce an asynchronous supply of glucose and amino acids to the animals. This asynchronous situation in LP diets may compromise amino acid utilization efficiency: 1) more amino acids will be consumed in the first-pass metabolism [4]; 2) efficiency of amino acid used for protein synthesis declines owing to the relative shortage of energy in the short term after a meal [5]; 3) systemic oxidation of amino acids increases [6]. Therefore, optimizing the dietary glucose release profile to promote the synchronous supply of glucose and amino acids may be a potentially feasible approach to improve the amino acid utilization efficiency of LP diets.

Starch is the major glucose source in feed. Compared with amylose, the amylopectin with branched form induces greater gelatinization, a greater surface area for digestive enzymes, and faster digestion and absorption (in the form of glucose) rates [7]. However, some studies have pointed out that the crystal structure, particle size, and physical form of starch also determine its digestion characteristics [8]. In this study, based on the ratio of amylopectin and amylopectin, and other factors were considered, LP diets with different glucose release properties were formulated to modulate the synchronism of glucose and amino acids supply.

Besides, previous research proved that nitrogen efficiency and nutrient utilization of livestock and poultry were also greatly affected by posterior intestinal microbial flora and its fermentation products [9]. Starch itself is the substrate for microbial fermentation, can directly regulate the bacterial community and short-chain fatty acids (SCFAs) manufacture [10]. Also, the difference in starch digestion rate can cause the change in dietary protein digestion, and then indirectly influence hindgut microbial protein fermentation [11]. Therefore, it is valuable to investigate the influence of starch patterns in LP diets on the intestinal flora and SCFAs manufacture.

To sum up, the objective of the present study was to explore the effect of dietary starch patterns on protein deposition, nutrient metabolism, and microbial community, and investigate the optimal dietary glucose release profile for nitrogen efficiency and growth performance in growing pigs under LP diets condition.

## Materials and methods

Animal experiments were approved by the China Agricultural University Animal Care and Use Committee (Beijing, AW21101202-1-2).

### General

All pigs and experimental supplies were provided by the Fengning Swine Research Unit of China Agricultural University (Academician Workstation in Chengdejiuyun Agricultural and Livestock Co., Ltd). Experimental diets were formulated based on the National Research Council [12] and our previous research [1], with slight modifications (Table 1).

**Table 1:** Ingredients and nutrient composition of experimental diets (as-fed basis)

Items	W LP	C + W LP	C LP	P + W LP	P LP
Ingredient, %					
Waxy corn starch^1^	57.91	28.95	–	28.95	–
Corn starch^2^	–	28.95	57.91	–	–
Field pea starch^3^	–	–	–	28.95	57.91
Soybean meal	20.68	20.68	20.68	20.68	20.68
Wheat bran	8.00	8.00	8.00	8.00	8.00
Alfalfa meal	6.00	6.00	6.00	6.00	6.00
Cellulose acetate	2.00	2.00	2.00	2.00	2.00
Dicalcium phosphate	1.93	1.93	1.93	1.93	1.93
Limestone	0.22	0.22	0.22	0.22	0.22
Salt	0.30	0.30	0.30	0.30	0.30
Premix^4^	1.00	1.00	1.00	1.00	1.00
L-Lysine	0.53	0.53	0.53	0.53	0.53
DL-Methionine	0.25	0.25	0.25	0.25	0.25
L-Tryptophan	0.04	0.04	0.04	0.04	0.04
L-Threonine	0.23	0.23	0.23	0.23	0.23
L-Valine	0.24	0.24	0.24	0.24	0.24
L-Leucine	0.29	0.29	0.29	0.29	0.29
L-Isoleucine	0.10	0.10	0.10	0.10	0.10
L-Phenylalanine	0.16	0.16	0.16	0.16	0.16
L-Histidine	0.12	0.12	0.12	0.12	0.12
Calculated composition					
Net energy, kcal/kg	2550	2550	2550	2550	2550
SID Lys, %	1.01	1.01	1.01	1.01	1.01
SID SAA, %	0.58	0.58	0.58	0.58	0.58
SID Trp, %	0.18	0.18	0.18	0.18	0.18
SID Thr, %	0.63	0.63	0.63	0.63	0.63
SID Val, %	0.63	0.63	0.63	0.63	0.63
SID Leu, %	1.03	1.03	1.03	1.03	1.03
SID Ile, %	0.57	0.57	0.57	0.57	0.57
SID Phe, %	0.59	0.59	0.59	0.59	0.59
SID His, %	0.34	0.34	0.34	0.34	0.34
SID Arg, %	0.69	0.69	0.69	0.69	0.69
Analyzed composition, %					
Dry matter	89.78	89.43	90.01	89.60	88.98
Crude protein	13.00	13.10	13.31	13.08	13.25
Crude fiber	3.75	3.81	3.77	3.82	3.75
Neutral detergent fiber	8.21	8.08	8.14	8.11	8.21
Acid detergent fiber	4.89	4.91	4.78	7.78	5.00
Total calcium	0.67	0.66	0.67	0.64	0.63
Total phosphorus	0.55	0.58	0.55	0.54	0.57
Total starch, g/kg	656.05	649.38	660.27	658.43	662.28
Amylose, g/kg	30.50	110.44	187.25	191.01	283.32
Amylopectin^5^, g/kg	625.55	538.94	473.02	467.42	378.96
Amylopectin/Amylose ratio	20.51	4.88	2.53	2.45	1.33

^1^ Waxy corn starch were purchased from Shandong Fuyang Biological Starch Co., Ltd., with a starch purity of 99%

^2^ Corn starch were purchased from Shandong Fuyang Biological Starch Co., Ltd., with a starch purity of 99%

^3^ Filed pea starch were purchased from Shandong Shandong Maidaofeng Cereals and Oils Co., Ltd., with a starch purity of 99%

^4^ Premix provided the following per kg of complete diet for growing pigs: vitamin A, 5512 IU; vitamin D_3_, 2200 IU; vitamin E, 64 IU; vitamin K_3_, 2.2 mg; vitamin B_12_, 27.6 μg; riboflavin, 5.5 mg; pantothenic acid, 13.8 mg; niacin, 30.3 mg; choline chloride, 551 mg; Mn, 40 mg (MnSO_4_); Fe, 100 mg (FeSO_4_∙H_2_O); Zn, 100 mg (ZnSO_4_); Cu, 100 mg (CuSO_4_∙5H_2_O); I, 0.3 mg (KI); Se, 0.3 mg (Na_2_SeO_3_)

^5^ Amylopectin content = total starch content - amylose content

*W LP* waxy corn starch low-protein diet, *C + W LP* corn starch + waxy corn starch low-protein diet, *C LP* corn starch low-protein diet, *P + W LP* pea starch + waxy corn starch low-protein diet, *P LP* pea starch low-protein diet, *SID* standardized ileal digestibility

### Nitrogen metabolism and protein deposition

Thirty crossbred (Duroc × Landrace × Yorkshire) growing barrows (initial weight: 29.7 ± 2.0 kg) were allotted randomly to a completely randomized design with 5 dietary treatments. The experimental diets were LP semi-homogeneous diets (crude protein: 13%) composed of purified starch and feedstuffs mainly included soybean meal, wheat bran, alfalfa meal and crystalline amino acids. According to the dietary starch patterns, the experimental treatments were divided into: (1) a 100% waxy corn starch LP (W LP); (2) a 50% corn starch and 50% waxy corn starch LP (C + W LP); (3) a 100% corn starch LP (C LP); (4) a 50% pea starch and 50% waxy corn starch LP (P + W LP) and (5) a 100% pea starch LP (P LP). Pigs were housed individually in stainless-steel metabolism crates (1.4 m × 0.7 m × 0.6 m). Daily feed allotment (about 4% of body weight; BW) was divided into two equal meals given to each pig at 09:00 and 16:00 during the first 23-d experimental period. All the pigs had access to fresh water ad libitum. The temperature was maintained at 23 ± 2 °C. Humidity varied from 55% to 65% during the experiment.

The experiment lasted for 37 d. Before samples collection, pigs were acclimated to metabolism crates for 7 d and fed a standard corn-soybean meal diet and then were fed experimental diets for another 7 d to adapted to the diet. During the adaptation period, we trained pigs to finish each meal within 10 min. Feces and urine were collected from each pig on d 15 through d 19. Feces were collected into plastic bags immediately as they appeared in the metabolism crates and stored at − 20 °C. Urine was collected into a bucket located under the metabolism crates. The bucket contained HCl (10 mL; 6 mol/L) to limit microorganism multiplication and reduce the loss of ammonia. A 10% aliquot of urine was filtered through gauze and immediately stored at − 20 °C. At the end of the 5-d collection, the feces and urine were pooled respectively for each pig and the feces subsamples (about 250 g) were dried for 72 h at 65 °C and ground through a 1-mm screen. Feces and urine samples were stored at 4 °C and − 20 °C respectively for analysis.

On the morning of d 20, 150 mg (about 5 mg/kg BW) [^15^N] glycine packed in a starch capsule was provided to each pig. Urine excreted from d 20 to d 22 was collected and stored at − 20 °C until protein turnover analysis.

### Growth performance

Barrows were weighed after an overnight fast at d 23 and continued to be fed the same treatment diets as previous but with ad libitum access. We fed the pigs three times a day at 08:00, 14:00, and 20:00. Because pigs maintained the rapid-ingesting habit, pigs can finish eating within 15 min after each feeding. During the 14-d growth experiment period, the feed intake of each pig was recorded daily. On the afternoon of d 36, fresh feces (about 2 g) from each pig were collected into two sterile centrifuge tubes (5-mL) and then stored at − 20 °C after quick-freezing by liquid nitrogen. On the morning of d 37, after an overnight fast, blood sample collection was conducted on each pig from the jugular vein into an anti-coagulant tube (Becton, Dickinson and Company, Franklin Lakes, NJ, USA). Pigs were weighed after blood collection to determine average daily gain (ADG) and feed efficiency.

### Chemical composition analysis

Chemical analyses of feed, feces, and urine samples were conducted in duplicate. Concentrations of dry matter (930.15) and crude protein (954.01) in feed, urine, and feces samples were measured according to the Association of Official Analytical Chemists procedures (2000) [13]. Concentrations of crude fiber (978.10), total calcium (984.01), and total phosphorus (965.17) in feed and feces samples were measured according to the Association of Official Analytical Chemists procedures (2006) [14]. Acid detergent fiber and neutral detergent fiber in feed ingredients were determine by using a fiber analyzer according to a previous report [15]. Total starch and amylose contents were determined with commercial assay kits (Nanjing Jiancheng Bioengineering Institute, Nanjing, China).

### Starch in vitro digestion

The in vitro digestion of dietary starch was determined in quadruplicate as described by Englyst et al. with minor modifications [16]. In brief, samples (about 1 g) were incubated in a HCl solution (10 mL; 0.05 mol/L) containing 0.05 g pepsin (P-7000; Sigma-Aldrich) and 0.05 g guar gum (P-9000-30-0; Sigma-Aldrich). Tubes were incubated at 37 °C for 30 min with vibration. Then, sodium acetate solution (10 mL; 0.25 mol/L) and a mixed enzyme digestive juice [5 mL; 0.7 g pancreatin (P-7545; Sigma-Aldrich, Darmstadt, Germany), 0.05 mL amyloglucosidase, and 3 mg invertase (P-57629; Sigma-Aldrich, Darmstadt, Germany)] was added to each tube. After incubating at 37 °C for 0.25, 0.50, 1, 1.5, 2, 3, and 4 h, an aliquot of 0.5 mL was taken respectively to which absolute ethanol was added for stopping the digestion of the starch. Then the samples were centrifuged at 3000 × *g* for 10 min to get supernatant. The glucose content of the supernatant was measured using a commercial glucose detection kit (Nanjing Jiancheng Bioengineering Institute, Nanjing, China).

### Plasma analysis

The plasma free amino acid concentration was determined as follow. Frozen serum samples (1 mL) were placed at 4 °C to thaw and then deproteinized with salicylic acid (120 mg). After the samples were placed in an ice bath for 25 min, the reaction system was centrifuged (12,000 × *g* for 15 min) and then the supernatant was adjusted to pH 7.0 by adding a lithium hydroxide solution (2 mol/L). The supernatant fluid was filtered through a filter (0.1 μm) before amino acid analysis using an S-433D Amino Acid Analyzer (Sykam, Munich, Germany).

Plasma SCFAs concentrations were determined according to a previous study [17], with minor modifications. In brief, an aliquot of plasma (0.4 mL) was added into one sterile centrifuge tube (2 mL) containing acetonitrile (1.2 mL). The solution was centrifuged at 12,000 × *g* for 15 min. Afterward, the supernatant was diluted five times with ultrapure water and then used for SCFAs analysis using a gas chromatography (Ion Chromatography System; Dionex ICS-3000, Sunnyvale, CA, USA).

The concentrations of plasma pyruvate, insulin, leptin, aldolase, insulin-like growth factor-1 (IGF-1), and lipase were determined using Enzyme-Linked Immunosorbent Assay (ELISA) methods with commercial kits (Sinoukbio Biotechnology Institute, Beijing, China). The determination of the plasma levels of alanine aminotransferase, aspartate aminotransferase, alkaline phosphatase, creatinine, total bilirubin, glucose, glycogenic amino acids, total protein, albumin, urea nitrogen, total cholesterol, triglyceride, low-density lipoprotein, high-density, and lipoprotein was conducted using colorimetry methods with commercial kits (Beijing Winter Song Boye Biotechnology Co. Ltd., Beijing, China). ELISA determination was operated using an automatic enzyme label analyzer (DR-200BS, Huawei Langde instrument Co., Ltd., Wuxi, China). Colorimetry determination was operated using an automatic biochemical analyzer (BS-420, Mindary Biomedical Electronics Co., Ltd., Shenzhen, China). All the determinations were conducted according to the manufacturers’ protocols.

### Lactate and SCFAs concentrations in feces

Lactate and SCFAs concentrations in feces were analyzed as described previously [17]. In brief, thawed fecal samples (about 1 g) were placed into sterile tubes (10 mL), diluted with ultrapure water (8 mL) and homogenized. Afterward, samples were incubated in an ultrasonic bath for 30 min then centrifuged at 10,000 × *g* for 10 min to obtain the supernatant. Concentrations of lactate and SCFAs in the supernatant were measured using gas chromatography (Ion Chromatography System; Dionex ICS-3000, Sunnyvale, CA, USA).

### Whole-body protein turnover

Urinary urea and ammonia were determined in two subsamples using commercial kits (Nanjing Jiancheng Bioengineering Institute, Nanjing, China). ^15^N analysis was conducted according to a previous study [18]. In brief, for urea and ammonia separation from total urinary nitrogen, a cation exchange resin (AG50W-X8 200 H^+^ form; Sigma-Aldrich, St. Louis, MO, USA) was converted to the Na^+^/K^+^ form by stirring of resin (100 g) twice for 15 min each in NaOH (600 mL; 0.1 mol/L). The resin was then washed to neutrality with Milli-Q water (18.2 MΩ, Millipore, Billerica, MA, USA) and stirred three times for 15 min each in Na^+^/K^+^ phosphate buffer (600 mL; 0.2 mol/L) adjusted to pH 7.4.

To extract the urinary ammonia nitrogen, an aliquot of urine (containing approximately 3000 μg of ammonia) was passed through the Na^+^/K^+^ resin to combine the ammonia, and then ammonia was washed from the Na^+^/K^+^ resin. The ammonia was stored at − 20 °C for ^15^N enrichment analysis. The elute containing the urea fraction was subjected to enzymatic hydrolysis (Urease Type VI Jack Beans, 105,000 units/g, Sigma-Aldrich Co.) to obtain ammonia. All the ammonia was washed from the resin and transferred into a ‘Y’ volumetric flask connected to a lithium hypobromite reaction liquid. Finally, ammonia, derived from urea or present in the urine, was detected by automated mass spectrometry (MM 903: VG Isogas, Middlewich, Cheshire, UK).

### Bacterial community structure

Feces samples in W LP, C LP, and P LP treatments were detected for the bacterial community. Because, these three groups can respectively represent the high, middle, and low starch digestion rate groups of the present study, we did not analyze bacterial community in C + W LP and P + W LP treatments, which is considered unnecessary and complicated. A QIAamp Fast DNA Stool Mini Kit (Qiagen Ltd., Düsseldorf, Germany) was used for bacterial DNA extraction according to the manufacturer’s instructions. The agarose gel electrophoresis proved that DNA isolation was achieved as expected. A thermocycler polymerase chain reaction system (GeneAmp 9700, ABI, USA) was applied to amplify the bacterial 16S rRNA genes in the V3–V4 hypervariable region. And the Illumina HiSeq 2500 platform (San Diego, CA, USA) was used to purify, quantify, pool, and sequence the resulting amplicons. Within Qiime (version 1.8; http://qiime.org/), the sequences were clustered into OTUs with a 97% similarity. Then, UCHIME was used for the definition and removal of the nonnormal gene sequences. The Ribosomal Database Project classifier (http://rdp.cme.msu.edu/) was also referenced to perform a taxon-dependent analysis of OTUs at a 90% confidence level. The α and β diversity analysis and taxonomic community assessments were conducted using Qiime 1.8 scripts.

### Calculation

The feed efficiency was calculated using the following formula:

1 $$ \mathrm{Feed}\ \mathrm{efficiency}=\mathrm{total}\ \mathrm{feed}\ \mathrm{intake}/\mathrm{total}\ \mathrm{weight}\ \mathrm{gain} $$

The starch digestion coefficient was calculated as follow:

2 $$ \mathrm{Digestion}\ \mathrm{coefficient}\ \mathrm{at}\ \mathrm{t}\mathrm{ime}\ \mathrm{t}=\left[\left(\mathrm{glucose}\ \mathrm{present}\ \mathrm{at}\ \mathrm{t}\mathrm{ime}\ \mathrm{t}-0\ \min\ \mathrm{glucose}\ \mathrm{release}\right)\times 0.9\right]/\mathrm{total}\ \mathrm{starch} $$

0.9 Is the molecular weight of glucose as incorporated into starch

The dietary amylose content was calculated as follow:

3 $$ \mathrm{Dietary}\ \mathrm{amylopectin}\ \mathrm{content}=\mathrm{total}\ \mathrm{starch}\ \mathrm{content}-\mathrm{amylose}\ \mathrm{content} $$

The apparent biological value was calculated as follow:

4 $$ \mathrm{Apparent}\ \mathrm{biological}\ \mathrm{value}=\left(\mathrm{intake}\ \mathrm{of}\ \mathrm{nitrogen}-\mathrm{fecal}\ \mathrm{nitrogen}-\mathrm{urinary}\ \mathrm{nitrogen}\right)/\left(\mathrm{intake}\ \mathrm{of}\ \mathrm{nitrogen}-\mathrm{fecal}\ \mathrm{nitrogen}\right) $$

The net protein utilization was calculated as follow:

5 $$ \mathrm{Protein}\ \mathrm{efficiency}\ \mathrm{of}\ \mathrm{utilization}=\left(\mathrm{intake}\ \mathrm{of}\ \mathrm{nitrogen}-\mathrm{fecal}\ \mathrm{nitrogen}-\mathrm{urinary}\ \mathrm{nitrogen}\right)/\mathrm{intake}\ \mathrm{of}\ \mathrm{nitrogen} $$

Whole-body protein turnover was calculated according to the end-product method using a single oral dose of ^15^N glycine [19]. This simplified model is based on assumptions that ^15^N is distributed between protein synthesis and amino acid oxidation in the same proportion as total body amino acids; ^15^N released from protein degradation will not be reincorporated into protein; ^15^N is freely available for transamination between amino acids.

The rate of whole-body nitrogen flux was estimated using the equation of Waterlow et al. [19]:

6 $$ Q={E}_{\mathrm{EP}}\times d/{e}_{EP} $$

In this equation, *E*_*EP*_ referred to the amount of nitrogen excreted as urinary ammonia or urea (g/d); *d* is the dose of ^15^N given orally (g); *e*_*EP*_ is the total amount of ^15^N excreted in urine as ammonia or urea (g) during the collection period and *Q* is total whole-body nitrogen flux (g/d).

Absolute rates of whole-body protein synthesis (g of protein synthesized/d) and breakdown (g of protein degraded/d) were derived from the following equation:

7 $$ Q=I+B=S+E $$

In this equation, *I* is the sum of the digestible nitrogen intake from the diets and infused nitrogen, *B* is the rate of whole-body protein degradation, *S* is the rate of whole-body protein synthesis and *E* is total urinary N excretion (urinary urea plus ammonia), all expressed as g/kg BW^0.75^/d.

### Statistical analysis

The PROC MIXED procedures of SAS version 9.4 (SAS Institute, Cary, NC, USA) was used to perform date analysis. All data were checked normal distribution and homogeneous variance using the UNIVARIATE procedure. Data for growth performance, nitrogen metabolism and plasma free amino acid concentrations were analyzed using the ANOVA of SAS. Data for protein deposition, plasma biochemical parameters, lactate and SCFAs analysis was performed by the *t*-test procedure of SAS. Dietary treatment was a fixed effect and pig was the random effects in the model. Each pig was regarded as a repeat, so each treatment is repeated 6 times. Data obtained by ANOVA are shown as the Lsmeans and SEM, and data obtained by *t*-test are presented as means ± SD. Differences at *P*-value ≤ 0.01 were considered highly significant; differences at *P*-value ≤ 0.05 were considered significant and differences at *P*-value < 0.10 was considered a tendency.

The α diversity of the fecal bacterial community was analyzed using Mann-Whitney U test and Kruskal-Wallis test. The adonis to determine the statistical significance of the principal co-ordinates analysis (PCoA) analysis of microbial compositions between the treatments was done using QIIME software package (version 2) and was based on the Bray-curtis distance metrics. Linear discriminant analysis effect size (LEfSe) was used to compare differences in taxonomic levels, including phylum, class, order, family, and genus.

## Results

### Starch composition affected in vitro digestion rate

**Fig. 1:**
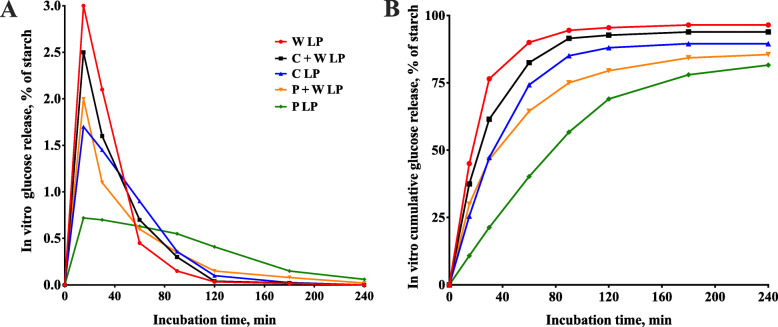
Dietary starch digestion and glucose release in in vitro experiment. Absolute (**A**) and cumulative (**B**) release of glucose during in vitro digestion were determined at 15, 30, 60, 90, 120, 180 and 240 min, respectively. Values are means, *n* = 4. In A, the SE ranged from 0.03% to 0.05%; in B, the SE ranged from 0.06% to 2.83%. Abbreviations: W LP, waxy corn starch low-protein diet; C + W LP, corn starch + waxy corn starch low-protein diet; C LP, corn starch low-protein diet; P + W LP, pea starch + waxy corn starch low-protein diet; P LP, pea starch low-protein diet

Starch digestion curves exhibited a lag phase that could be described with the sigmoidal shape parameter (Fig. 1 and Table. 2). In vitro glucose release peaked at 15 min after the start of incubation and the peak glucose release was 3.01%, 2.52%, 1.78%, 2.04%, and 0.72% for W LP, C + W LP, C LP, P + W LP, and P LP treatments, respectively. When at 120 min of incubation, glucose release was 0.03%, 0.04%, 0.10%, 0.15%, and 0.41% for the five treatments (Fig. 1A). Cumulative starch digestion at 30 and 240 min was 76.50% and 96.50% for W LP, 61.50% and 93.90% for C + W LP, 47.25% and 89.55% for C LP, 45.50% and 85.50% for P + W LP, and 10.80% and 81.60% for P LP, respectively (Fig. 1B).

**Table 2:** Dietary starch digestion and glucose release in vitro experiment

Items	W LP	C + W LP	C LP	P + W LP	P LP
In vitro glucose release of starch, %					
15 min	3.00	2.50	1.70	2.00	0.72
30 min	2.10	1.60	1.45	1.10	0.70
60 min	0.45	0.70	0.90	0.60	0.63
90 min	0.15	0.30	0.36	0.35	0.55
120 min	0.03	0.04	0.10	0.15	0.41
180 min	0.02	0.02	0.03	0.08	0.15
240 min	0.00	0.00	0.00	0.02	0.06
In vitro cumulative glucose release of starch, %					
15 min	45.00	37.50	25.50	30.00	10.80
30 min	76.50	61.50	47.25	46.50	21.30
60 min	90.00	82.50	74.25	64.50	40.20
90 min	94.50	91.50	85.05	75.00	56.70
120 min	95.50	92.70	88.05	79.50	69.00
180 min	96.50	93.90	89.55	84.30	78.00
240 min	96.50	93.90	89.55	85.50	81.60

*W LP* waxy corn starch low-protein diet, *C + W LP* corn starch + waxy corn starch low-protein diet, *C LP* corn starch low-protein diet, *P + W LP* pea starch + waxy corn starch low-protein diet, *P LP* pea starch low-protein diet

### Starch composition modulated growth performance

Daily weight gain of pigs fed C + W LP diet (952 g/d; Table 3) was greater than those fed P + W LP (738 g/d; *P* < 0.05) and P LP diets (790 g/d; *P* < 0.05). Feed efficiency of C + W LP group (0.48) tended to improve compared with that of P + W LP group (0.41; *P* = 0.08).

**Table 3:** Growth performance condition

Items	W LP	W + C LP	C LP	W + P LP	P LP	SEM	*P*-value
Initial weight, kg	35.4	35.6	35.5	35.4	35.5	2.4	0.994
Final weight, kg	46.7	48.9	47.0	45.7	45.8	2.8	0.782
Average daily gain, g	816^ab^	952^a^	823^ab^	738^b^	790^b^	50	0.041
Average daily intake, g	1882	1970	1886	1795	1789	91	0.308
Feed efficiency	0.43	0.48	0.44	0.41	0.44	0.03	0.379

^a-b^Means in the same row with different superscripts differ (*P* ≤ 0.05)

*W LP* waxy corn starch low-protein diet, *C + W LP* corn starch + waxy corn starch low-protein diet, *C LP* corn starch low-protein diet, *P + W LP* pea starch + waxy corn starch low-protein diet, *P LP* pea starch low-protein diet. Values are means of 6 observations per treatment

### Starch composition influenced nitrogen efficiency

C + W LP treatment showed significantly decreased total nitrogen excretion compared with the other treatments (*P* < 0.05; Table 4) and markedly decreased urinary nitrogen excretion and the ratio of urinary nitrogen to total nitrogen excretion compared with that of W LP, C LP, and P LP treatments (*P* < 0.05). Compared with pigs fed W LP and C LP diets, pigs fed C + W LP diet showed a significantly lower ratio of urinary nitrogen to total nitrogen excretion (*P* < 0.05), a higher ratio of fecal nitrogen to total nitrogen excretion (*P* < 0.05), and a higher apparent biological value (*P* < 0.05).

**Table 4:** Nitrogen balance of pigs in response to low-protein diets

Items	W LP	C + W LP	C LP	P + W LP	P LP	SEM^1^	*P*-value
Average daily feed intake, g/d	1040	1067	1070	1035	1048	35	0.941
Dietary N intake, g/d	21.64	22.36	22.78	21.66	22.22	1.41	0.947
Urinary N excretion, g/d	2.05^a^	1.37^b^	2.17^a^	1.61^ab^	1.91^a^	0.13	0.018
Fecal N excretion, g/d	4.26	4.25	4.39	4.73	4.50	0.25	0.743
Total N excretion, g/d	6.31^a^	5.62^b^	6.56^a^	6.34^a^	6.41^a^	0.20	0.046
Retention of N, g/d	15.33	16.74	16.22	15.32	15.81	0.78	0.342
Total N excretion/intake of N, %	29.17	25.13	28.80	29.26	28.84	1.75	0.390
Urinary N excretion/N excretion, %	32.48^a^	24.37^b^	33.08^a^	25.39^ab^	29.80^ab^	2.12	0.050
Fecal N excretion/N excretion, %	67.52^b^	75.63^a^	66.92^b^	74.61^ab^	70.20^ab^	2.42	0.050
Apparent biological value^2^, %	88.20^b^	92.44^a^	88.20^b^	90.49^ab^	89.22^ab^	1.25	0.043
Protein efficiency of utilization^3^, %	70.79	74.76	71.16	70.86	71.30	3.42	0.269

^a-c^ Means in the same row with different superscripts differ (*P* ≤ 0.05)

^1^*n* = 6

^2^ (Intake of nitrogen - fecal nitrogen - urinary nitrogen) / (intake of nitrogen - fecal nitrogen)

^3^ (Intake of nitrogen - fecal nitrogen - urinary nitrogen) / intake of nitrogen

*W LP* waxy corn starch low-protein diet, *C + W LP* corn starch + waxy corn starch low-protein diet, *C LP* corn starch low-protein diet, *P + W LP* pea starch + waxy corn starch low-protein diet, *P LP* pea starch low-protein diet. Values are means of 6 observations per treatment

### Starch composition affected plasma free amino acids

Compared with the other treatments, P + W LP treatment showed the lowest plasma asparagine, glutamate, methionine, glycine, and total non-essential amino acid concentrations and the highest plasma histidine concentration among treatments (*P* < 0.05; Table 5). W LP treatment showed markedly higher plasma alanine concentration than the other treatments (*P* < 0.05) Compared with the other treatment groups, the C + W LP group showed highly significantly lower plasma citrulline concentration (*P* < 0.01).

**Table 5:** Plasma free amino acid concentrations of growing pigs fed low-protein diets

Items	W LP	C + W LP	C LP	P + W LP	P LP	SEM^1^	*P*-value
Essential amino acids, μmol/L							
Lysine	193.75	186.80	192.99	184.23	193.29	13.59	0.761
Methionine	38.27^a^	39.85^a^	40.79^a^	22.73^b^	34.30^a^	3.21	0.038
Threonine	134.37^ab^	112.11^b^	156.53^a^	169.01^a^	120.61^b^	9.10	0.050
Tryptophan	53.89	55.99	54.04	58.42	50.14	4.88	0.662
Isoleucine	143.83	130.90	164.25	157.92	148.31	13.91	0.295
Valine	167.45	130.89	135.64	170.67	151.29	15.77	0.193
Arginine	187.40^a^	138.29^b^	147.71^ab^	138.25^b^	129.40^b^	14.20	< 0.001
Histidine	92.25^ab^	74.46^b^	69.74^b^	111.68^a^	73.87^b^	8.01	0.048
Phenylalanine	93.44	88.92	90.69	84.81	93.02	5.51	0.526
Total EAA	1104	958	1052	1097	994	65	0.440
Non-essential amino acids, μmol/L							
Aspartic acid	74.46	69.13	72.64	75.41	73.52	4.51	0.214
Glutamine	318.44	328.10	310.45	324.92	322.20	14.39	0.588
Glutamate	146.15^ab^	154.90^ab^	195.08^a^	132.51^b^	175.78^ab^	18.20	0.045
Alanine	194.03^a^	107.76^b^	126.14^b^	108.56^b^	129.69^b^	10.46	0.018
Cystine	66.28^a^	46.84^b^	51.31^b^	57.93^ab^	51.15^b^	4.52	0.044
Glycine	473.45^a^	439.48^a^	427.28^a^	309.02^b^	417.49^a^	22.91	0.008
Proline	142.50	126.59	134.49	121.52	133.99	10.77	0.523
Serine	86.62	78.29	89.62	82.62	92.62	6.76	0.146
Tyrosine	173.33	166.77	166.65	173.13	165.69	12.65	0.291
Taurine	74.76^abc^	78.56^ab^	85.96^a^	79.96^ab^	62.28^c^	5.27	0.021
Asparagine	168.53^ab^	178.60^a^	165.93^ab^	115.43^c^	125.53^bc^	15.73	0.008
Citrulline	71.52^a^	55.35^b^	73.03^a^	78.15^a^	78.25^a^	2.78	< 0.001
Ornithine	134.00	129.12	141.52	135.64	144.87	7.09	0.553
Total NEAA	2176^a^	2010^abc^	2101^ab^	1854^c^	2030^abc^	81	0.027
Total AA, μmol/L	3280	2969	3150	2952	3024	135	0.674

^a-c^ Means in the same row with different superscripts differ (*P* ≤ 0.05)

^1^*n* = 6

*W LP* waxy corn starch low-protein diet, *C + W LP* corn starch + waxy corn starch low-protein diet, *C LP* corn starch low-protein diet, *P + W LP* pea starch + waxy corn starch low-protein diet, *P LP* pea starch low-protein diet, *NEAA* non-essential amino acid. Values are means of 6 observations per treatment

### Starch composition modulated protein turnover

**Fig. 2:**
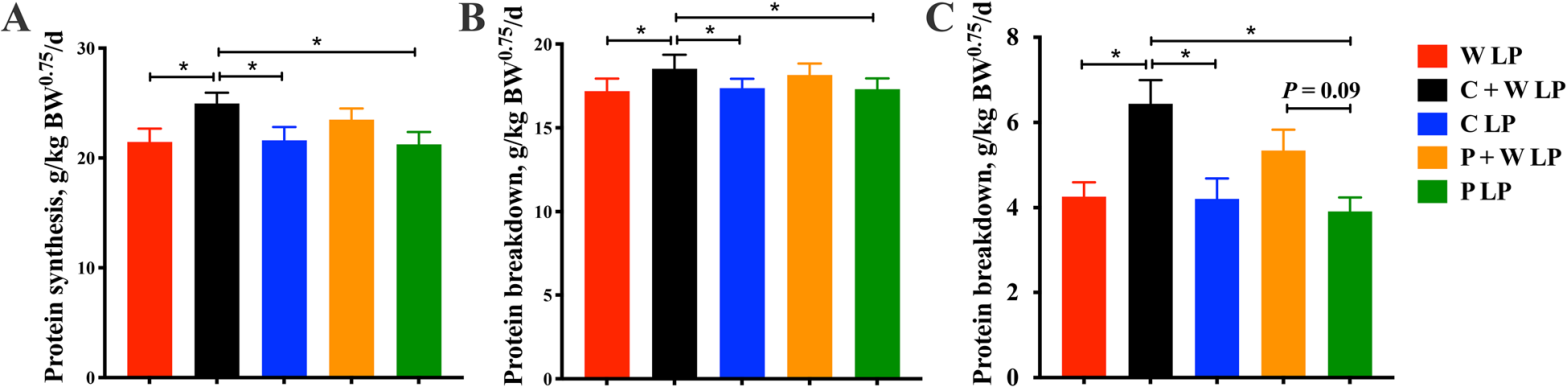
Protein turnover of growing pigs fed low-protein diets. Protein synthesis (**A**), protein breakdown (**B**) and protein deposition (**C**). Values are means of 6 observations per treatment. ^*^Significant differences between treatments (*P* ≤ 0.05). Values are least squares means ± SD; *n* = 6. Abbreviations: W LP, waxy corn starch low-protein diet; C + W LP, corn starch + waxy corn starch low-protein diet; C LP, corn starch low-protein diet; P + W LP, pea starch + waxy corn starch low-protein diet; P LP, pea starch low-protein diet

Protein synthesis, protein breakdown, and protein deposition in C + W LP group were dramatically increased compared with those in W LP, C LP, and P LP treatment groups (*P* < 0.05; Fig. 2). There was no significant difference between C + W LP and P + W LP treatments on protein synthesis, protein breakdown, and protein deposition. Protein deposition in the P + W LP group was numerically higher than that in the P LP group, but the difference was not significant.

### Starch composition acted on liver function and nutrient metabolism

**Fig. 3:**
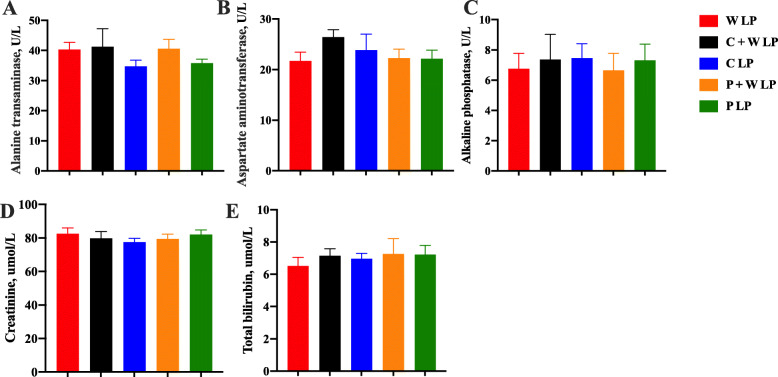
Liver function of growing pigs fed low-protein diets. Alanine aminotransferase (**A**), aspartate aminotransferase (**B**), alkaline phosphatase (**C**), creatinine (**D**), and total bilirubin (**E**). Values are means of 6 observations per treatment. Values are least squares means ± SD; *n* = 6. Abbreviations: W LP, waxy corn starch low-protein diet; C + W LP, corn starch + waxy corn starch low-protein diet; C LP, corn starch low-protein diet; P + W LP, pea starch + waxy corn starch low-protein diet; P LP, pea starch low-protein diet

**Fig. 4:**
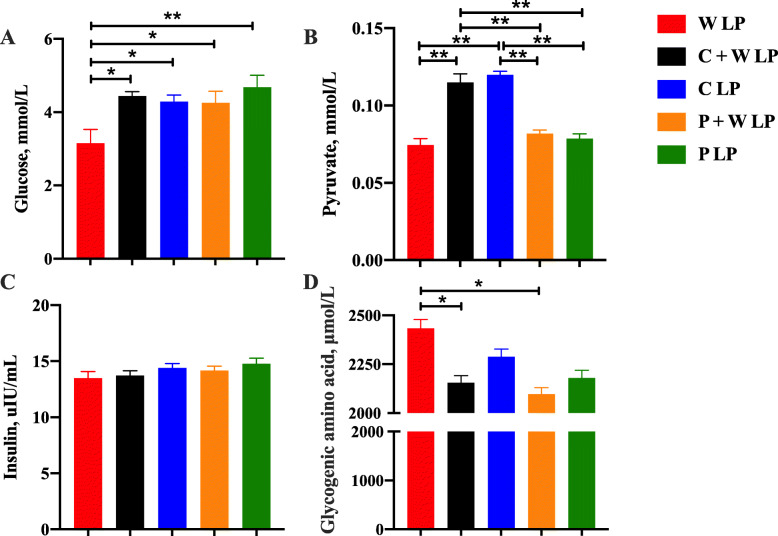
Carbohydrate metabolism of growing pigs fed low-protein diets. Glucose (**A**), pyruvate (**B**), insulin(**C**) and glycogenic amino acid (**D**). Values are means of 6 observations per treatment. ^*^Significant differences between treatments (*P* ≤ 0.05), ^**^Significant differences between treatments (*P* ≤ 0.01). Values are least squares means ± SD; *n* = 6. Abbreviations: W LP, waxy corn starch low-protein diet; C + W LP, corn starch + waxy corn starch low-protein diet; C LP, corn starch low-protein diet; P + W LP, pea starch + waxy corn starch low-protein diet; P LP, pea starch low-protein diet

**Fig. 5:**
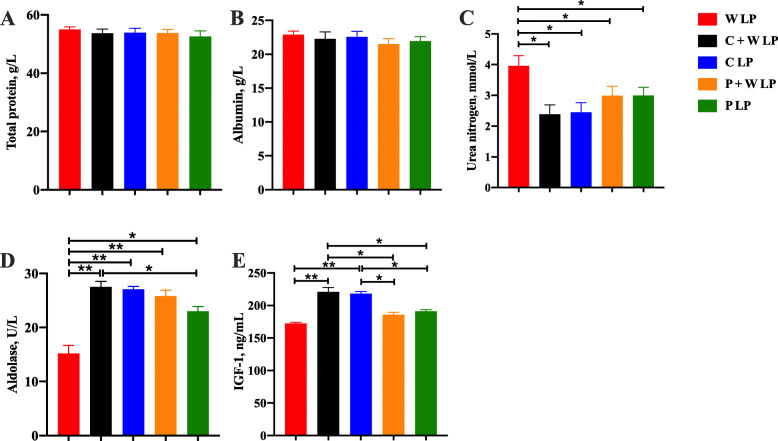
Nitrogen metabolism of growing pigs fed low-protein diets. Total protein (**A**), albumin (**B**), urea nitrogen (**C**), aldolase (**D**) and IGF-1 (**E**). Values are means of 6 observations per treatment. ^*^Significant differences between treatments (*P* ≤ 0.05), ^**^Significant differences between treatments (*P* ≤ 0.01). Values are least squares means ± SD; *n* = 6. Abbreviations: W LP, waxy corn starch low-protein diet; C + W LP, corn starch + waxy corn starch low-protein diet; C LP, corn starch low-protein diet; P + W LP, pea starch + waxy corn starch low-protein diet; P LP, pea starch low-protein diet

**Fig. 6:**
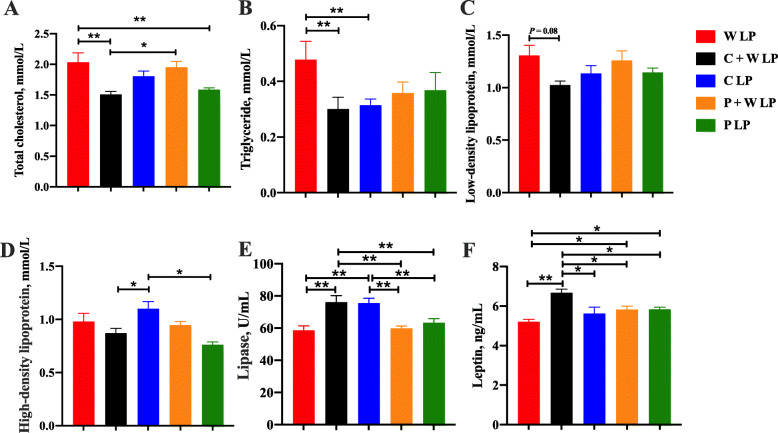
Lipid metabolism of growing pigs fed low-protein diets. Total cholesterol (**A**), triglyceride (**B**), low-density lipoprotein (**C**), high-density lipoprotein (**D**), lipase (**E**) and leptin (**F**). Values are means of 6 observations per treatment. ^*^Significant differences between treatments (*P* ≤ 0.05), ^**^Significant differences between treatments (*P* ≤ 0.01). Values are least squares means ± SD; *n* = 6. Abbreviations: W LP, waxy corn starch low-protein diet; C + W LP, corn starch + waxy corn starch low-protein diet; C LP, corn starch low-protein diet; P + W LP, pea starch + waxy corn starch low-protein diet; P LP, pea starch low-protein diet

Plasma alanine aminotransferase, aspartate aminotransferase, alkaline phosphatase, creatinine, and total bilirubin concentrations among treatments were not significantly different (Fig. 3). W LP group showed significantly lower plasma glucose and aldolase concentrations and higher plasma urea nitrogen concentration compared with the other groups (*P* < 0.05; Fig. 4 and Fig. 5). Compared with W LP, P + W LP, and P LP treatments, the plasma pyruvate concentrations in C + W LP and C LP treatments were dramatically increased (*P* < 0.01). Plasma total glycogenic amino acid concentration of pigs fed W LP diet was significantly higher than those of pigs fed C + W LP and P + W LP diets (*P* < 0.05). Plasma IGF-1 concentrations in C + W LP and C LP treatment groups did not differ but were notably higher than those in the other treatment groups (*P* < 0.05). There was no difference in plasma leptin concentration between treatments. The plasma total cholesterol concentration markedly increased in W LP treatment compared with those in C + W LP and P LP treatments (*P* < 0.05; Fig. 6). C + W LP and C LP treatment groups showed a highly significantly decreased plasma concentration of triglyceride compared with the W LP treatment group (*P* < 0.01). Plasma lipase concentrations in C + W LP and C LP treatment groups did not differ but were both notably higher than those in the other treatment groups (*P* < 0.05).

### Starch composition acted on SCFAs in plasma and feces

**Fig. 7:**
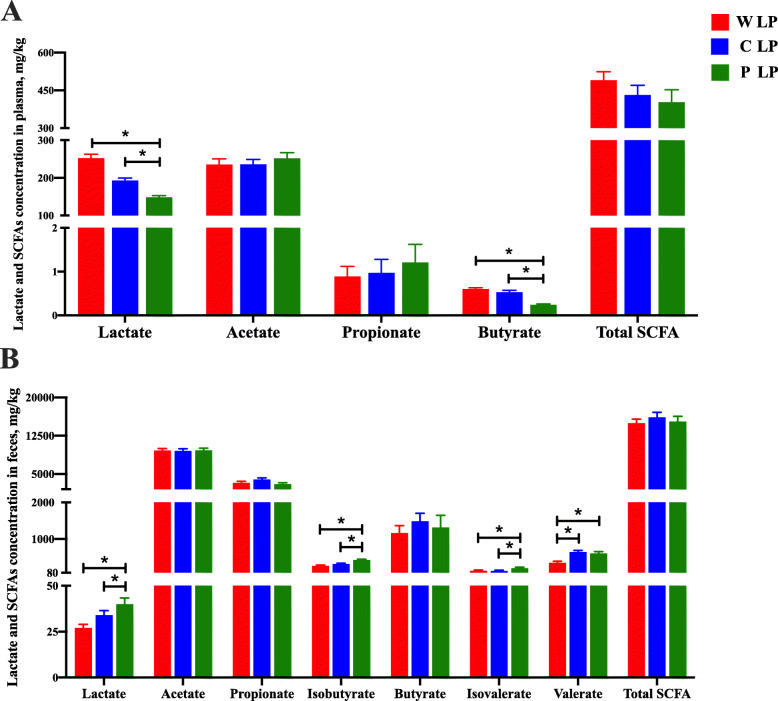
Plasma and feces lactate and SCFAs concentrations. Lactate and SCFAs concentrations in plasma (**A**) and feces (**B**). Values are means of 6 observations per treatment. ^*^Significant differences between treatments (*P* ≤ 0.05), ^**^Significant differences between treatments (*P* ≤ 0.01). Values are least squares means ± SD; *n* = 6. Abbreviations: W LP, waxy corn starch low-protein diet; C + W LP, corn starch + waxy corn starch low-protein diet; C LP, corn starch low-protein diet; P + W LP, pea starch + waxy corn starch low-protein diet; P LP, pea starch low-protein diet

Compared to the other treatment groups, the P LP group presented significantly decreased plasma lactate and butyrate concentrations (*P* < 0.05; Fig. 7). The concentrations of isobutyrate and isovalerate in feces of pigs fed P + W LP and P LP diets were markedly increased compared with those in pigs fed W LP, C + W LP, and C LP diets (*P* < 0.05). Besides, P + W LP and P LP groups presented significantly higher fecal lactate concentration than W LP and C + W LP groups (*P* < 0.05). Fecal valerate concentration of pigs fed C LP, P + W LP, and P LP diets were significantly increased compared with those of pigs fed W LP and C + W LP diets (*P* < 0.05).

### Starch composition modulated the fecal bacterial community

**Fig. 8:**
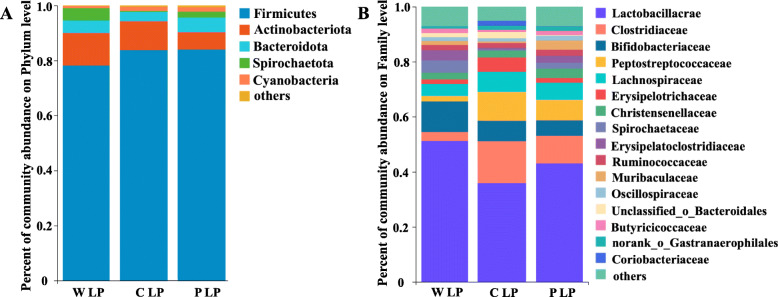
Fecal bacterial community at the phylum and family levels in growing pigs fed low-protein diet. Microbial community bar plot of phyla with an abundance of 0.015% or greater (**A**), and microbial community bar plot of families with a proportion of 0.015% or higher (**B**)

There was no significant difference in diversity and richness of bacterial community among the different treatments (Table 6). A phylum-level analysis proved that the microbiota composition in the feces of pigs was consistently dominated by Firmicutes (81.98%; Fig. 8) and Actinobacteriota (9.59%). At the family level, Lactobacillaceae (43.38%), Clostridiaceae (9.48%) and Bifidobacteriaceae (8.09%) were the dominant bacteria.

**Table 6:** The α diversity of the fecal bacterial community

Items	W LP	C LP	P LP	*P*-value
Shannon^1^	2.37	2.36	2.67	0.644
Simpson^2^	0.31	0.27	0.21	0.801
ACE^3^	273.80	266.31	284.40	0.766
Chao^4^	276.50	273.65	288.89	0.814

^1^ Shannon-Wiener index, an index to measure species diversity: an increase in the Shannon-Wiener index value represents an increase in species diversity

^2^ Simpson index, describes the probability that the number of individuals sampled from a community twice in a row belong to the same species

^3^ Abundance-based Coverage Estimator, is used to estimate the number of OTUs contained in the community and is one of the commonly used indices for estimating the total number of species in ecology

^4^ Chao index, is commonly used in ecology to estimate the total number of species

*W LP* waxy corn starch low-protein diet, *C + W LP* corn starch + waxy corn starch low-protein diet, *C LP* corn starch low-protein diet, *P + W LP* pea starch + waxy corn starch low-protein diet, *P LP* pea starch low-protein diet. Values are means of 6 observations per treatment

**Fig. 9:**
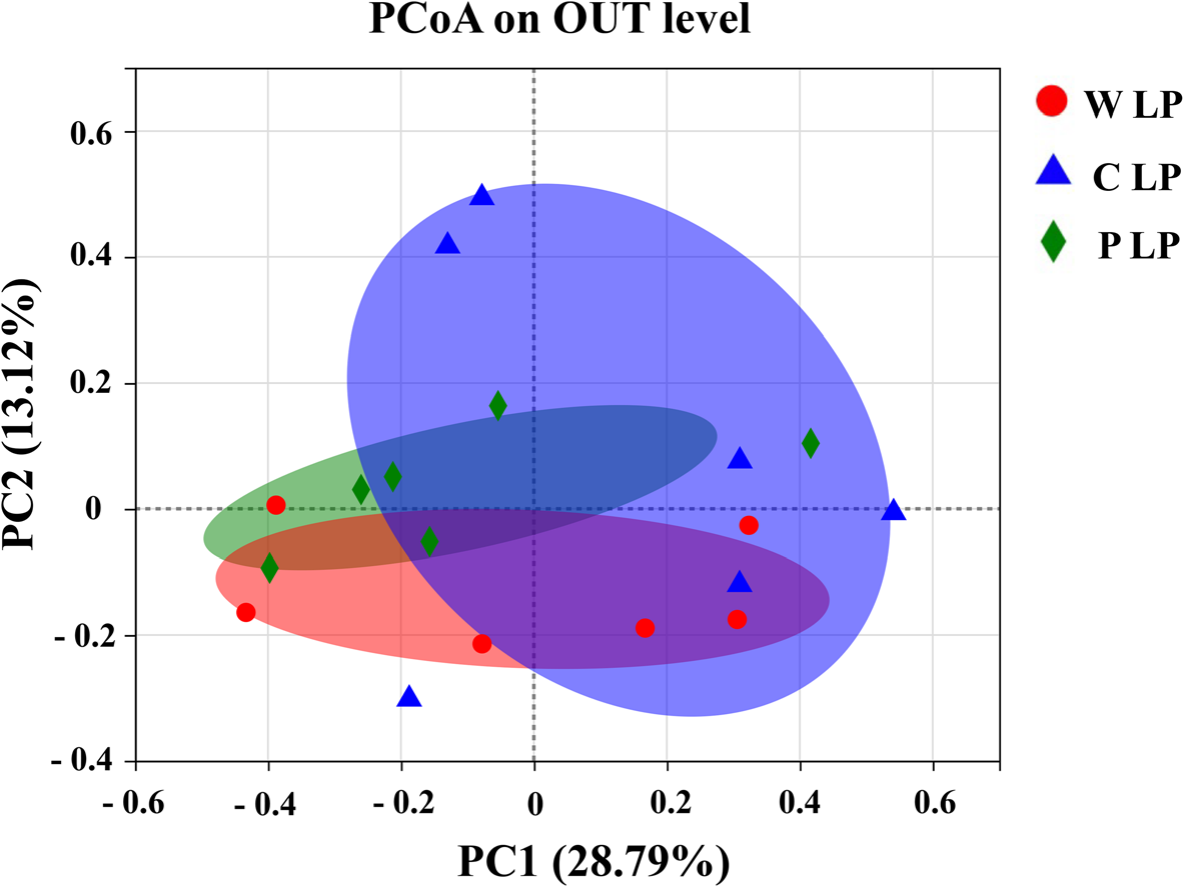
Principal coordinate analysis of the microbiota from feces of pigs fed low-protein diet. Principal coordinate analysis of treatment groups based on Bray-curtis analyses of bacterial communities at the operational taxonomic unit level. The distances between the symbols on the ordination plot reflect the relative dissimilarities in the community structures

To analyze the microbial communities, a binary Pearson distance matrix was constructed based on the OTUs in each sample of treatments. The PCoA revealed that starch patterns (in W LP and P LP treatments) in LP diets changed the taxonomic and functional structures of the microbial communities, respectively (*P* < 0.05; Fig. 9).

**Fig. 10:**
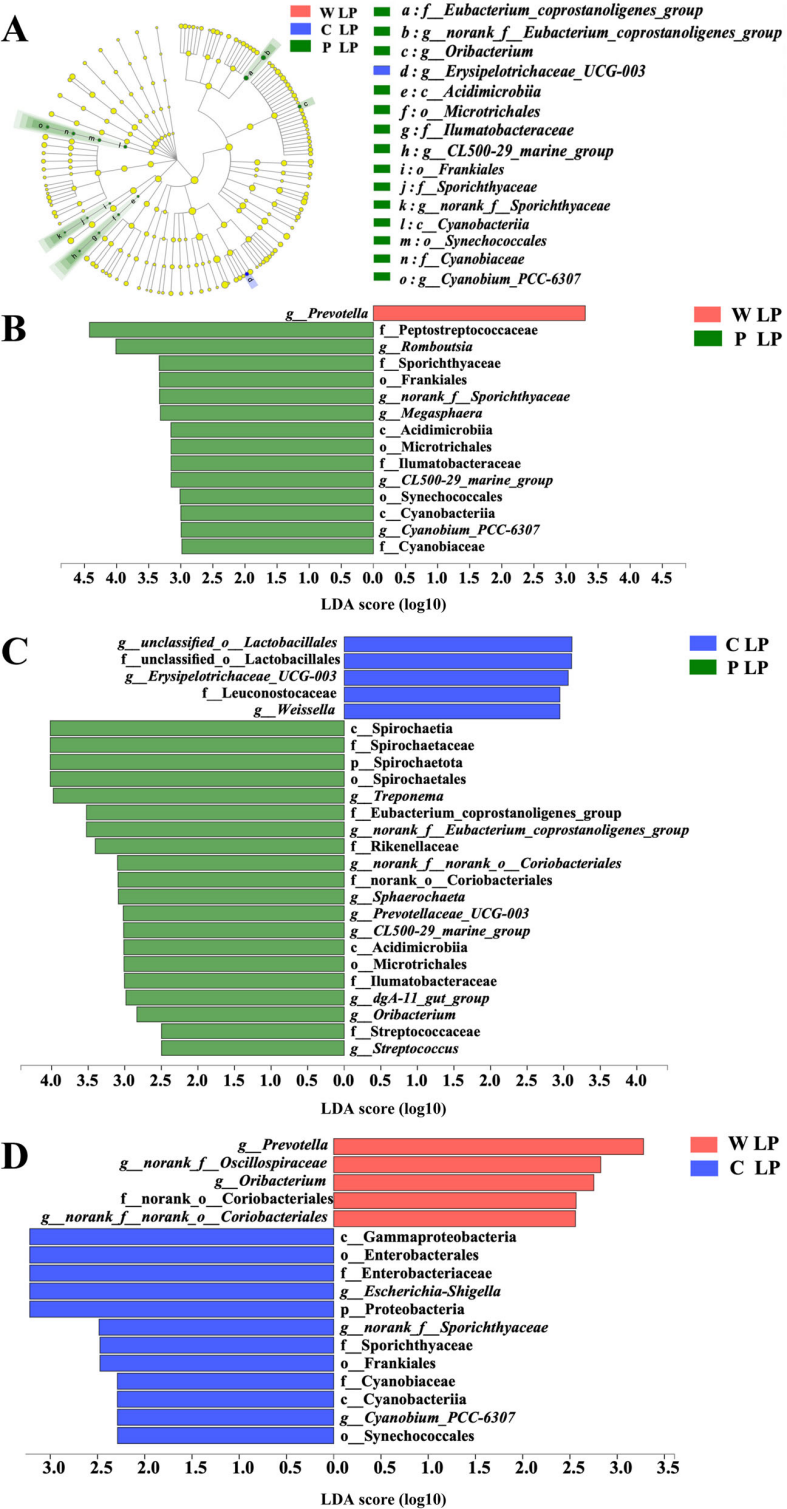
LEfSe effect size results of the microbiota in fecal samples. Histogram of the linear discriminant analysis scores computed for the differentially abundant features in the fecal bacteria between W LP, C LP and P LP treatments (**A**), W LP and P LP treatments (**B**), C LP and P LP treatments (**C**) and W LP and C LP treatments (**D**). The linear discriminant analysis bars indicate the microbial groups within treatments with linear discriminant analysis scores higher than 2.0. The differentially abundant clades in each treatment are represented by colors in the cladograms, and the linear discriminant analysis scores of these clades indicate the degrees of statistical and biological differences

Significant differences in the microbial community among the different treatment groups are shown in Fig. 10. Many bacteria such as Eubacterium*_*Coprostanoligenes*_*Group, Romboutsia, Sporichthyaceae, Oribacterium, and *Cyanobium_PCC-6307* were in greater abundance in the P LP treatment than W LP and C LP treatments. Compared with the P LP treatment, the W LP treatment had an increased abundance of *Prevotella* and a decreased abundance of Peptostreptococcaceae, Cyanobacteria, and Ilumatobacteraceae.

## Discussion

By supplementing crystalline amino acids, LP diets can precisely satisfy the nutritional requirement of livestock and poultry while saving feed costs and reducing nitrogen pollution excretion. But crystalline amino acids supplementation can also have negative effects. Crystalline amino acids release amino acids faster than starch releases glucose in the intestine. Therefore, the absorption and metabolism of amino acids occur earlier than that of glucose in pigs fed LP diets supplemented with large amounts of crystalline amino acids. This may cause a decrease in amino acid efficiency for the following reasons. 1) Owing to the lack of non-essential amino acids such as glutamine, asparagine, and glutamate, and glucose supply lagging behind amino acids supply, crystalline amino acids are oxidized to provide energy for intestinal cells under the fuel-shortage conditions [6, 20, 21]. 2) Previous studies proved that the postprandial concentration peak of blood amino acids caused by crystalline amino acids ingestion was markedly earlier than that of blood glucose caused by corn starch ingestion [22, 23]. This will cause glucose to be unable to provide sufficient energy during the process of amino acid synthesis into protein, compromising protein synthesis [24]. 3) The increase in postprandial blood glucose concentration stimulates the secretion of hormones that benefit protein synthesis (such as leptin, IGF-1, and insulin) [25, 26]. Therefore, the uncoordinated supply of glucose and amino acids also weakens the protein synthesis promotion from hormones. 4) Large amounts of amino acids that cannot be synthesized into protein are in free form, stimulating the body to oxidize these amino acids to avoid toxicity [5]. Hence, enhancing the synchronization of glucose and amino acids supply in LP diets is extremely important for efficient amino acid utilization.

Starch, the main glucose source in diet and feed, is a critical metabolism and health regulator. Previous studies suggested that slowly-digested starch usually rich in amylose, such as tubers and legume, [27] can delay digestion, nourish microorganisms to produce SCFAs, and benefit colonic health [28]. Besides, waxy starches rich in amylopectin are more efficient in enzymatic hydrolysis, which leads to higher digestibility and faster digestion rate [29], resulting in greater postprandial blood glucose and related hormones fluctuation and more food consumption [30]. Therefore, dietary starch patterns modulation may be a feasible method to optimize nitrogen efficiency and regulate nutrient metabolism in LP diets. In the current study, the ratio of dietary amylopectin to amylose was used as the main factor regulating the rate of glucose release from starch. The results of the present study demonstrated that dietary starch patterns modulation can regulate nutrient metabolism, protein turnover, and microbial fermentation, and the optimal dietary glucose release profile can enhance protein deposition and improve nitrogen efficiency and growth performance in growing pigs fed LP diets.

Waxy corn starch is almost entirely composed of amylopectin, while corn starch and pea starch contain about 25% and 45% amylose, respectively. The results of in vitro digestion experiments demonstrated that we successfully regulated the glucose release rate of the diets with starch patterns modulation. It is worth noting that although the C LP group and the P + W LP group had a similar ratio of amylopectin to amylose, the in vitro glucose release patterns were significantly different. This was expected because, as we stated earlier, starch digestion rates are regulated by other factors such as particle size. Compared with the C LP diet, the waxy corn starch in the P + W LP diet promoted a rapid glucose release in the first 15 min, and the pea starch caused a sustained glucose release after 90 min. The present study did not research in vivo postprandial change in blood glucose with time after a meal, because our previous data [31] and van Kempen et al. [32] has confirmed that the in vitro digestion rate of starch can reflect the rate of postprandial change in blood glucose in growing pigs, approximately.

Protein turnover is a basic physiological process of organisms, including protein synthesis and degradation. The balance of protein synthesis and degradation is a decisive factor affecting protein deposition in animal cells and tissues (such as skeletal muscle) [18]. Protein turnover cannot conduct independently of energy metabolism. The ATP consumption to synthesize or degrade 1 mol of protein in the cell is 5 mol and 1 mol, respectively. Previous studies found that when under maintenance states, the protein synthesis rate of a 30 kg growing pig is 270 g/d. And when the dietary energy value increased to 2 and 3 times the maintenance energy requirement, this rate increased to 406 g/d and 512 g/d, respectively [33]. Few studies focused on the effect of energy carrier supply rate on protein turnover. Results of the present study demonstrated that when waxy corn starch was used to moderately increase the glucose release rate of LP diets composed of corn starch or pea starch, the increase in protein synthesis was greater than that in protein degradation, and then protein deposition was effectively enhanced. Crystalline amino acids in LP diets are rapidly absorbed by the gut after meals, whereas intact proteins are digested to release amino acids and then absorbed. In C + W LP diets, waxy corn starch high in amylopectin can release glucose rapidly, and corn starch releases glucose relatively continuously at a moderate rate. Therefore, the C + W LP diet may have provided the pigs with a simultaneous supply of amino acids and glucose, which facilitated protein synthesis from amino acids.

The results of nitrogen efficiency were consistent with those of protein turnover. There was no significant difference in fecal nitrogen excretion among treatments, which indicated that dietary starch patterns did not affect the total tract digestibility of protein. Urine nitrogen refers to the nitrogen that is absorbed into the body and excreted after being metabolized [34]. Significant differences were observed in urine nitrogen excretion between treatment groups. The similarity in fecal nitrogen excretion combined with differences in urine nitrogen between treatments indicates that starch patterns changes altered the utilization efficiency of post-absorptive nitrogen. Especially, the C + W LP group significantly improved the protein apparent biological value compared with the C LP group. This may be related to the fact that waxy corn starch accelerated postprandial glucose release from the diet, improved the synchronization of glucose and amino acids supply in LP diets. The enhanced nitrogen efficiency contributed to the improved daily gain of pigs in the C + W LP group.

The gastrointestinal digestion of amylopectin is faster than that of amylose, which results in that pigs on high amylopectin diets absorbing glucose more rapidly after meals [35, 36]. Given that a rapid increase in blood glucose in a short time can stimulate the massive secretion of insulin for negative feedback [29], decreased plasma glucose concentration in the W LP treatment may be because the rapidly absorbed glucose is converted into glycogen by the massively secreted insulin. Glycogenic amino acids refer to amino acids that can be converted into glucose participating in energy metabolism [37]. The increased plasma concentrations of glycogenic amino acids, such as alanine and arginine, may indicate an increased higher amino acid catabolism to furnish glucose in the W LP group. Pyruvate can be reduced to lactate in the cytoplasm for energy, enter the mitochondria to participate in aerobic respiration, and achieve the transformation of three major nutrients through the acetyl-CoA and tricarboxylic acid cycle [38]. The increased plasma pyruvate concentrations in the C + W LP and C LP groups represented relative energy metabolism homeostasis.

Enhanced muscle protein synthesis is accompanied by increased plasma IGF-1 concentration, and plasma urea nitrogen reflects systemic amino acid oxidation [39, 40]. In the present study, the decreased IGF-1 concentration and increased urea nitrogen concentration in the W LP treatment indicated that excessively rapid glucose release rate in LP diets compromised muscle protein synthesis and increased amino acid oxidation. Elevated plasma aldolase concentration has been used to report adult muscular dystrophy and muscle breakdown [41]. But for growing animals, enhanced muscle protein synthesis is generally accompanied by strengthened muscle protein breakdown (the whole-body protein turnover data in the present study confirmed this) [18]. Hence, the lowest plasma aldolase concentration in W LP treatment was probably related to compromised muscle protein synthesis. Leptin is a circulating hormone produced by obesity genes [42]. Generally, fat deposition promotes leptin secretion; obesity leads to elevated fasting plasma leptin concentrations. In the present study, there was no significant difference in the plasma leptin concentration of growing pigs among the treatment groups, indicating that the fat deposition was similar among treatments. Previous studies have suggested that rapidly digested starch can cause obesity, contradicting the results of the present experiment [43, 44]. This may be because rapidly digested starch in LP diets improve the synchronization of amino acid and glucose digestion and absorption, leading to enhanced protein synthesis. Large amounts of glucose are used to fuel protein synthesis, which reduces energy deposited in the form of fat.

Total cholesterol and triglycerides are the main components of blood lipids. Compared with the C + W LP group, increased plasma total cholesterol and triglyceride concentrations in W LP may be associated with decreased lipase and leptin concentrations, which can promote fat catabolism [45, 46].

Since the gut microbial flora and its metabolites have an important impact on the nutrient utilization of the host [8], we explored the effects of three different purified starches on pig feces SCFAs and flora structure under LP conditions. Results suggested that the fecal microbial community and metabolites were markedly modulated by the dietary starch patterns. Compared with the W LP and C LP treatment groups, the P LP treatment group showed an increased abundance of the *Eubacterium Coprostanoligenes* Group. Coprostanoligenes can convert cholesterol to coprostanol, which is absorbed poorly by the gastrointestinal system. Previous studies found that Coprostanoligenes effectively reduced the content of serum cholesterol in rats and rabbits [47, 48]. This is consistent with our research results that Coprostanoligenes enriched in the P LP treatment group with a decreased plasma cholesterol concentration. Besides, in the P LP treatment, Romboutsia was prosperous. Romboutsia can make massive proliferation in nutrient-rich environments [49]. Results of our other research proved that the P LP treatment decreased ileal digestibility of total amino acids (69% vs. 78% and 80%) and starch (92% vs. 98% and 98%) compared with the W LP and C LP treatment. The sufficient nutrients entering in hindgut may have caused the prosperity of Romboutsia in the P LP group in the present study. In addition, it is worth noting that the rich nutrients in the hindgut also nourish many harmful bacteria in the P LP treatment, such as *Cyanobium_PCC-6307*, a potentially toxic bacteria that exists in water [50]; *Oribacterium*, a kind of Lachnospiraceae, enriched in early colorectal cancer [51]; Sporichthyaceae, a potential disease indicator for sea bass [52]. On the opposite, the W LP diet is rich in amylopectin that can be quickly digested by the intestine, which reduces the amounts of nutrients entering the hind intestine. W LP was rich in Prevotella, which can relieve inflammation and modulate caspase-8-mediated IL-1β maturity [53].

Lactate is one of the major end products of probiotic carbohydrate fermentation [54]. More ileal indigestible starch in the P LP diet is fermented by microorganisms to produce more lactate, which causes the lactate content in feces to be increased [29]. The decreased starch digestion efficiency can also hinder the chemical digestion of dietary protein in the foregut, resulting in more protein into the hindgut for microbial fermentation. The increased fecal isobutyrate and isovalerate (branched-chain fatty acids, microbial protein fermentation products) contents in P LP treatment confirmed this [55]. Previous studies found that fasting plasma lactate concentration was elevated in obese subjects with metabolic syndrome compared to healthy lean individuals, which may be owing to an impairment in aerobic metabolism [56, 57]. The significantly decreased plasma lactate concentration in the P LP treatment may characterize strong aerobic metabolism capacity. Another interesting finding is that the slowly digested starch (P LP diet) in this study reduced the fasting plasma butyrate concentration, which is contrary to a previous study that suggested Indigestible carbohydrates increase plasma butyrate the next morning [58]. The difference in experimental methods (fasting or not) may be one of the reasons for this difference.

## Conclusion

Dietary starch patterns modulation can regulate nutrient metabolism, protein turnover, and fecal microbial fermentation in pigs. The dietary glucose release profile can be effectively adjusted by changes in dietary starch patterns. In C + W LP diets, waxy corn starch high in amylopectin can release glucose rapidly, and corn starch releases glucose relatively continuously at a moderate rate. Compared to other treatment diets, optimal dietary glucose release profile in C + W LP diets effectively strengthen whole-body protein deposition and improve nitrogen efficiency and growth performance in growing pigs fed LP diets.

## Data Availability

The data analyzed during the current study are available from the corresponding author on reasonable request.

## Abbreviations

ADG: Average daily gain
BW: Body weight
ELISA: Enzyme-linked immunosorbent assay
IGF-1: Insulin-like growth factor-1
LP: Low-protein
SCFAs: Short-chain fatty acids
SID: Standardized ileal digestibility
